# Pattern of Local Recurrence After Conservative Surgery and Radiotherapy for Soft Tissue Sarcoma

**DOI:** 10.1155/S1357714X01000160

**Published:** 2001-06

**Authors:** Susan J. Cleator, Chris Cottrill, Clive Harmer

**Affiliations:** 1 Sarcoma Unit Royal Marsden Hospital NHS Trust London SW3 6JJ UK; 2 St Bartholomew's Hospital London EC1A 7BE UK; 3 Department of Radiotherapy Royal Marsden Hospital Fulham Rd. London SW3 6JJ UK

## Abstract

*Purpose:* Over the past three decades our centre has adopted a policy of conservative surgery followed by adjuvant radicaldose radiotherapy for medium-and high-grade soft tissue sarcomas. For all cases of local recurrence following this treatment we aimed to define the spatial relationship between sites of recurrence and the positions of the phase 1 and 2 radiotherapy volumes.

*Patients:* We identified 25 cases of local recurrence recorded on our soft tissue sarcoma database between 1986 and 1999 inclusive. We excluded patients with macroscopic residual disease following surgery. Most patients were treated with a phase I volume corresponding to the entire muscle compartment (50 Gy in 25 fractions over 5 weeks) and a phase II volume corresponding to the tumour bed (10 Gy in five fractions). Six of the patients were treated according to a hyperfractionated regimen.

*Methods:* For each case we reviewed the diagnostic imaging, planning radiographs and prescription sheets. We audited whether treatment had been given according to protocol and defined whether recurrence had arisen in the phase 1 volume, phase 2 volume or ‘out of field’.

*Results:* Four (16%) patients recurred within the phase I volume, 17 (68%) recurred within the phase II volume and four (16%) outside the irradiated volume including one marginal recurrence. In six patients there had been deviation from our radiotherapy protocol (usually unavoidable) including all three true out of field recurrences.

*Discussion:* The majority of recurrences occur in the phase 2 volume. Prospective multi-centre data collection and, ideally, a prospective randomised trial are required to formulate an improved treatment policy with respect to radiotherapy margins and dose.


Sarcoma (2001) 5, 83–88

ORIGINAL ARTICLE

### Pattern of local recurrence after conservative surgery and radiotherapy for soft tissue sarcoma

SUSAN J. CLEATOR[1], CHRIS COTTRILL[2] & CLIVE HARMER[1]

[1]Sarcoma Unit, Royal Marsden Hospital NHS Trust, London SW3 6JJ, and [2]St Bartholomew's Hospital, London EC1A 7BE, UK

**Abstract**

*Purpose:* Over the past three decades our centre has adopted a policy of conservative surgery followed by adjuvant radical-dose radiotherapy for medium- and high-grade soft tissue sarcomas. For all cases of local recurrence following this treatment we aimed to define the spatial relationship between sites of recurrence and the positions of the phase 1 and 2 radiotherapy volumes.

*Patients:* We identified 25 cases of local recurrence recorded on our soft tissue sarcoma database between 1986 and 1999 inclusive. We excluded patients with macroscopic residual disease following surgery. Most patients were treated with a phase I volume corresponding to the entire muscle compartment (50 Gy in 25 fractions over 5 weeks) and a phase II volume corresponding to the tumour bed (10 Gy in five fractions). Six of the patients were treated according to a hyperfractionated regimen.

*Methods:* For each case we reviewed the diagnostic imaging, planning radiographs and prescription sheets. We audited whether treatment had been given according to protocol and defined whether recurrence had arisen in the phase 1 volume, phase 2 volume or 'out of field'.

*Results:* Four (16%) patients recurred within the phase I volume, 17 (68%) recurred within the phase II volume and four (16%) outside the irradiated volume including one marginal recurrence. In six patients there had been deviation from our radiotherapy protocol (usually unavoidable) including all three true out of field recurrences.

*Discussion:* The majority of recurrences occur in the phase 2 volume. Prospective multi-centre data collection and, ideally, a prospective randomised trial are required to formulate an improved treatment policy with respect to radiotherapy margins and dose.

**Key words:** *sarcoma, post-operative radiotherapy, recurrence, conservative surgery*

### Introduction

The recommended treatment of resectable high-grade soft tissue sarcoma is conservative, organ-preserving surgery followed by adjuvant radical radiotherapy. Combined modality treatment of this nature can achieve 5-year local control rates of 85–90%[1–7] and 5-year overall survival rates in excess of 70%.[1–3,5,7,8] In terms of local control and survival this compares favourably with the results achieved by radical surgery or amputation.[5,9,10] In addition to a local failure rate of up to 20% at 5 years, local recurrences later than this have been documented.[2] Approximately 60% of recurrences are salvageable with further surgery but this may involve amputation.[2–4]

In delivering postoperative radiotherapy we aim to improve functional outcome by reducing the extent of surgical resection required to achieve cure. However, radiotherapy morbidity can also impact on function and there is evidence that the risk of complications increases with both dose[7,8,11] and field size.[11] Between sarcoma units practice varies considerably with respect to the size of radiation portal employed relative to the tumour bed; some centres, including ours, irradiate the entire muscular compartment whilst others utilise a much smaller volume, treating the tumour bed with a margin of a few centimetres only by means of brachytherapy.[12]

Over the last two decades our unit has adopted a treatment policy of conservative surgery and adjuvant radiotherapy for all high and medium-grade tumours. The majority of patients are treated in accordance with a strict radiotherapy protocol.[13] Our sarcoma database was used to identify 25 cases of local recurrence dating back to 1986. Disease and treatment details relating to each case were analysed to identify the exact spatial relationship between site of recurrence and the irradiated volume.

Correspondence to: Dr Susan Cleator, Department of Radiotherapy, Royal Marsden Hospital, Fulham Rd., London SW3 6JJ, UK. Fax: +44-20-7808-2094; E-mail: suzy.cleator@virgin.net

1357–714X print/1369–1643 online/01/020083-06 © 2001 Taylor & Francis Ltd
DOI: 10.1080/13577140120048584

### Patients and methods

### *Patient and tumour characteristics (Tables 1 and 2)*

Since 1973 all new patients seen in our multidisciplinary sarcoma unit have been prospectively recorded on a database. This was used to identify patients who had demonstrated local relapse following conservative surgery and adjuvant radiotherapy. Planning and diagnostic radiographs were available for cases recorded since 1986 and hence our analysis dates back to this time. Patients with residual macroscopic disease following surgery or with metastatic disease (including nodal disease) at original presentation were excluded. Low-grade sarcomas were treated with postoperative radiotherapy only if they demonstrated multiple local recurrences or were associated with unresectable residual disease. The study was closed in 1999 resulting in a median follow-up time from completion of radiotherapy to time of writing of 58 months (range 16–150 months). Patients treated with preoperative radiotherapy have not been analysed. The patient and tumour characteristics are summarised in Table 1. All histology was reviewed in our centre by the same pathologist. The median age at presentation was 56 (range 17–86). Tumours arising in the limb and girdle comprised 88%, the remainder arising within the trunk. The percentage of tumours which were grade 1, 2 and 3 were 8, 32 and 60%, respectively. T1 tumours comprised 36% of patients and 64% were T2. Patients were staged according to the 1997 International Union Against Cancer (UICC) staging system.[14] Surgical margins were positive or 'probably positive' in nine patients (38%). In nine patients tumours were recurrent, having been previously treated by surgery alone. Four patients had metastatic disease at time of local recurrence. The total number of patients of this type treated over this period was 239, resulting in a local recurrence rate of 10.5%. The histological breakdown of the 239 patients is displayed in Table 2.

**Table 1.** *Recurrences, patient and tumour characteristics*

| Factor | | Number of patients | Percentage |
|---|---|---|---|
| *Age at diagnosis (years):* | | | |
| <30 | | 2 | 8 |
| 30–60 | | 14 | 56 |
| >60 | | 9 | 36 |
| Median | | 56 | |
| *Gender:* | | | |
| Male | | 13 | 52 |
| Female | | 12 | 48 |
| *Site:* | | | |
| Limb and girdle | | 22 | 88 |
| Trunk | | 3 | 12 |
| *Time to relapse (months):* | | | |
| Range | 3–86 | | |
| Median | 21 | | |
| *Histological type:* | | | |
| Liposarcoma | | 3 | |
| Leiomyosarcoma | | 8 | |
| Malignant fibrous histiocytoma | | 6 | |
| Synovial sarcoma | | 4 | |
| Dermatosarcoma protruberans | | 1 | |
| Fibrosarcoma | | 1 | |
| Unclassified high-grade sarcoma | | 2 | |
| *Grade:* | | | |
| 1 | | 2 | 8 |
| 2 | | 8 | 32 |
| 3 | | 15 | 60 |
| *Stage:* | | | |
| T1 (5 cm or less) | | 9 | 36 |
| T2 ( more than 5 cm) | | 16 | 64 |
| *Margins:* | | | |
| Positive | | 9 | 36 |
| Negative | | 16 | 64 |

### *Treatment details (Table 3)*

All patients were reviewed prior to treatment by the multidisciplinary team consisting of surgeon, radiologist, medical and clinical oncologist. Some patients underwent surgery in another institution and were referred for adjuvant treatment. When previous surgery was considered sub-optimal and where technically feasible, wide re-excision was performed. A comprehensive work-up included physical examination, preoperative MRI or CT scan of the region of disease and a CT scan of the lungs. All patients were treated under the supervision of a single radiotherapist.

Our standard radiotherapy treatment policy is to include the entire length of the involved muscle or muscle groups in the phase 1 planning target volume (PTV). Thereafter the PTV is reduced as a phase 2 consisting of the original tumour extent with a 2-cm margin. Where beneficial, treatment is CT-planned and wedges or remote tissue compensators are used to optimise the dose distribution. Customised casts

**Table 2.** *Histological profile of total patients treated*

| Histological types | Number (%) (*n* = 239) | Number of recurrences (%) |
|---|---|---|
| Leiomyosarcoma | 55 (23) | 8 (14.5) |
| Malignant fibrous histiocytoma | 52 (22) | 6 (11.5) |
| Liposarcoma | 37 (15) | 3 (8) |
| Synovial sarcoma | 33 (14) | 4 (12) |
| MPNST[a] | 13 (5) | 0 |
| Ewings | 6 (3) | 0 |
| Fibrosarcoma | 3 (1) | 1 (33) |
| Dermatofibrosarcoma protruberans | 2 (1) | 1 (50) |
| Others/unspecified[b] | 38 (16) | 2 |

[a]Malignant peripheral nerve sheath tumour.
[b]Clear cell sarcoma, chondrosarcoma, haemangiopericytoma, adult rhabdomyosarcoma, fibromatosis (two cases), epithelioid saracoma, angiosarcoma.

**Table 3.** *Treatment details*

| Case | Phase 1: Dose(Gy)/Number of fractions/frequency/ beam | Phase 2: Dose(Gy)/Number of fractions/frequency/ beam |
|---|---|---|
| 1 | 40/20/daily/photons | Not given |
| 2 | 46/23/daily/photons | Not given |
| 3 | 60/50/bd/photons | 12/10/bd/photons |
| 4 | 52.5/25/daily/photons | 7.5/5/daily/photons |
| 5 | 50/25/daily/photons | 10/5/daily/photons |
| 6 | 50/25/daily/photons | 10/5/daily/photons |
| 7 | 60/50/bd/photons | 12/10/bd/photons |
| 8 | 50/25/daily/photons | 12.5/5/daily/electrons |
| 9 | 50/25/daily/photons | 12.5/5/daily/electrons |
| 10 | 50/25/daily/electrons | 10/5/daily/electrons |
| 11 | 47/23/daily/photons | 13/5/daily/electrons |
| 12 | 50/25/daily/photons | 10/5/daily/photons |
| 13 | 50/25/daily/photons | 10/5/daily/photons |
| 14 | 60/50/bd/photons | 12Gy/10/bd/photons |
| 15 | 60/50/bd/photons | 12Gy/10/bd/photons |
| 16 | 50/25/daily/photons | 10Gy/5/daily/photons |
| 17 | 40/20/daily/photons | 20Gy/10/daily/photons |
| 18 | 50/25/daily/photons | 10Gy/5/daily/photons |
| 19 | 60/50/bd/photons | 12Gy/10/bd/photons |
| 20 | 50/25/daily/photons | 10Gy/5/daily/photons |
| 21 | 50/25/daily/photons | 10Gy/5/daily/photons |
| 22 | 50/25/daily/photons | 10Gy/5/daily/photons |
| 23 | 50/25/daily/photons | 10Gy/5/daily/photons |
| 24 | 60/50/bd/photons | 12Gy/10/bd/photons |
| 25 | 50/25/daily/photons | 10Gy/5/daily/photons |

are used to immobilise extremities and fields are shaped with lead blocks or cut-outs. Irradiation of the entire circumference of a limb is avoided, with care being taken to spare a corridor of skin and subcutaneous tissue. In both phases joints are spared as much as possible. When advantageous, three-dimensional planning and conformal radiotherapy with use of the multileaf collimator are utilised.[15]

Most patients were treated with high energy 5- or 6-MV photons alone, usually with parallel opposed beams which were sometimes angled. The phase 1 volume was treated to a dose of 50 Gy (to 100%) in 25 daily fractions over 5 weeks and the phase 2 volume to 10 Gy (to 100%) in five daily fractions during the sixth week. Where considered more appropriate, phase 2 was treated with an electron field. During the period analysed, a number of patients were treated in a study of hyperfractionated radiotherapy.[16] The planning protocol was unchanged but the phase 1 volume received 60Gy in 50 fractions of 1.2 Gy given twice daily over 5 weeks. The phase 2 volume then received a further 12 Gy in 10 twice daily fractions during the sixth week of treatment.

In each case of local recurrence we retrospectively reviewed diagnostic imaging, planning radiographs, portal films, prescription sheet diagrams and dosimetry details. We analysed firstly whether placement of the volumes had been appropriate and secondly the relationship between site of recurrence to the phase 1 and 2 volumes. Planning films were not available for three patients, but the relationship between the radiation field and origin of recurrence (phase 1, phase 2 or out of field) was deduced using the case notes and prescription sheet. In six cases the geographical origin of the recurrence was difficult to identify due to the recurrence being extensive or marginal (on the margin of one of the volumes). In five cases there was an obvious epicentre and the site of the recurrence was allocated accordingly. The remaining case was truly marginal. Portal images were present for most patients and in all cases lead protection to normal tissue had been positioned as prescribed.

### Results

### *Analysis of recurrence (Table 4)*

The commonest soft tissue sarcoma types seen were (in decreasing order of frequency): leiomyosarcoma, malignant fibrous histiocytoma, liposarcoma and synovial sarcoma. Numbers are too small to permit formal statistical analysis of variations in recurrence rate according to histological subtype.

The median time to local recurrence was 21 months (range 3–86 months) with eight (32%) occurring within 1 year and eight at or beyond 3 years. Two patients did not receive a radical dose. One had received 33 Gy at another hospital prior to surgery and received a further 40 Gy under our care, subsequently demonstrating recurrence on the margin of the phase 1 radiation field. The other patient (with an obturator internus leiomyosarcoma) stopped treatment at 46 Gy because of suspected intra-abdominal progression; no disease was found at laparotomy but an in-field recurrence occurred 22 months later. The remaining 23 patients received at least 60 Gy and have been divided into those with positive and those with negative pathological margins. A total of eight patients who received radical dose had positive margins or were thought to have a high risk of residual microscopic disease due to suboptimal surgery such as enucleation. An out-of-field relapse occurred in one patient who had undergone multiple excisions, laser vapourisations and skin grafting before radiotherapy; the radiation volume did not encompass all the previously grafted area because of concern regarding morbidity. The seven remaining patients received radiotherapy strictly according to protocol: six recurred in the phase 2 and one in the phase 1 volume (treated with hyperfractionation). One of the phase 2 recurrences had undergone enucleation only with further excision not having been undertaken due to complications associated with the initial surgery. Therefore, in this patient surgery had been suboptimal, but in the other six there were no identified technical causes for treatment failure.

There were 15 patients with clear histological margins who relapsed despite radical dose radiotherapy. One patient with liposarcoma of chest wall received

**Table 4.** *Detail of relapse*

| Case | Margin status | Site of relapse | Treatment factors possibly contributing to local relapse |
|---|---|---|---|
| 1 | Clear | Margin of phase 1 | Sub-optimal dose |
| 2 | Positive | Within phase 1 | Sub-optimal dose |
| 3 | Positive | Out of field | Whole of grafted area not covered |
| 4 | Positive | Within phase 2 | None |
| 5 | Positive | Within phase 2 (extensive) | None |
| 6 | Positive | Within phase 2 | Sub-optimal surgery (enucleation) |
| 7 | Positive | Within phase 1 | None |
| 8 | Positive | Within phase 2 | None |
| 9 | Positive | Within phase 2 | None |
| 10 | Positive | Within phase 2 | None |
| 11 | Clear | Within phase 1 | Phase 1 dose sub-optimal |
| 12 | Clear | Within phase 1 (extensive) | None |
| 13 | Clear | Out of field (extensive) | Muscle origin not covered |
| 14 | Clear | Within phase 2 | None |
| 15 | Clear | Within phase 2 | None |
| 16 | Clear | Out of field | Muscle origin not covered |
| 18 | Clear | Within phase 2 (extensive) | None |
| 19 | Clear | Within phase 2 (extensive) | None |
| 20 | Clear | Within phase 2 | None |
| 21 | Clear | Within phase 2 | None |
| 22 | Clear | Within phase 2 | None |
| 23 | Clear | Within phase 2 | None |
| 24 | Clear | Within phase 2 | None |
| 25 | Clear | Within phase 2 | None |

only 47 Gy to the phase 1 volume and relapsed within this volume. Another patient who was treated for a pleomorphic liposarcoma of the left triceps relapsed locally outside the volume; this treatment could be criticised for not having covered adequately the muscle origin. One patient relapsed following treatment for a grade 2 fibrosarcoma arising from the lower end of the psoas muscle; the radiation field did not cover the upper part of the pelvis due to risk of bowel toxicity; recurrence occurred above the proximal field margin. The remaining 12 cases conformed strictly to our protocol: 11 recurred in the phase 2 volume (four hyperfractionated) and one within the phase 1 volume.

### *Patient outcome*

With a median follow-up time since completing radiotherapy of 58 months, 10 patients have died. Nine deaths were disease-related and all died with metastatic disease. At time of writing, eight of the 15 survivors are alive and disease-free, although three have undergone pulmonary metastasectomy and four have required limb or, in one case, finger amputation. One patient has been lost to follow-up. Five survive with persistent local disease and one with metastatic disease.

### Discussion

The ultimate aim in the management of soft tissue sarcoma is to achieve local control and cure whilst ensuring organ preservation and limb function. The value of radiotherapy in reducing the incidence of local recurrence following conservative surgery has been demonstrated in historical series[1] and in two prospective randomised trials.[12,17] Debate continues in the literature as to whether improved local control translates to improved survival.[7,12] In retrospective studies local recurrence has been associated with a poorer survival. However, this may not be a causal relationship but rather an association that arises because locally recurrent tumours are biologically more aggressive.

Tumour-related factors shown to be associated with risk of local recurrence include high-grade histology,[4,8,12] size greater than 5 cm in diameter[2,8] and previous local recurrence.[3] In Pister's analysis of 1041 patients with extremity soft tissue sarcoma histological subtypes fibrosarcoma and malignant peripheral nerve tumour were significant independent adverse prognostic factors for local recurrence.[18]

With regard to treatment, presence of histologically positive margins, radiotherapy dose and radiotherapy margins have been analysed as possible factors influencing local control. Several retrospective series support the significance of positive operative margins in this respect.[5,6,8,18] Mundt[7] retrospectively reviewed 64 cases of soft tissue sarcoma treated by conservative surgery and adjuvant radiotherapy. Patients treated with an initial field margin beyond the tumour of $<5$ cm had a 5-year local control rate significantly worse than those treated with an initial field margin of $>5$ cm (30 vs. 93%, $p = 0.0003$). Fein[6] reviewed 67 patients (again retrospectively), and demonstrated significantly improved local control associated with a dose of ≥62.5 Gy compared with <62.5 Gy (95 vs. 78%). It is too early to assess the benefit of delivering radiotherapy in a hyperfractionated manner, although a phase 2 study in our centre demonstrated the regimen to be well tolerated with local control comparable to standard fractionation.[16]

Can analysis of our data tell us anything about the importance of margin status, the adequacy in size of the phase 1/2 volumes and the dose received to each? In six patients technical factors can be identified which may have accounted for failure, including all three out of field recurrences.

Eleven patients recurred inside the phase 2 volume despite negative margins, of whom four had been treated with the hyperfractionated regimen. Assuming an $\alpha/\beta$ ratio of 10 for tumour, this hyperfractionated regimen delivers an 11% increase in effective dose for tumour control relative to standard treatment.[16] A radiation dose-response for sarcoma cells has been demonstrated in experimental systems,[19] although prospective, randomised clinical data are lacking. Our adjuvant dose of 60 Gy is less than that applied in many centres where doses approach 70 Gy. It is possible that some of our local recurrences may have been avoided by delivery of a higher dose. Furthermore, work is required to determine if different doses should be applied to different sub-classes of sarcoma. In our centre treatment has always been given in two phases, the rationale being that the phase 2 volume requires a higher dose and because of concerns regarding the toxicity associated with the delivery of a full radical dose to a large phase 1 volume. However, any clonogenic cells remaining after surgery in the phase 1 volume will have inherent radiobiological properties identical to those remaining in the phase 2 volume, although there may be differences in the number of clonogenic cells and the tumour microenvironment, particularly oxygenation. A single-phase approach may be acceptable, particularly if more limited radiation field sizes are to be adopted.

Furthermore, it is conceivable that subsequent local relapse may have been prevented in margin positive cases by further surgery (where technically feasible) to achieve microscopically negative margins.

Treatment in six patients deviated from our radiotherapy protocol for reasons defined above. Of the 19 patients treated strictly according to protocol, two relapsed in the phase 1 volume and 17 in the phase 2 volume. Therefore, the majority of patients relapse at or very close to the tumour bed. On the basis of the results of this study it is possible to suggest that either treatment may be delivered as effectively with a 'limited field' or that 50 Gy is effective in treating microscopic disease within the phase 1 volume. Indeed the Memorial Sloan- Kettering group[12] achieved very good local control rates by irradiating the tumour bed with a margin of 2 cm only by means of brachytherapy supporting the former of these conclusions. The rationale of extending the irradiated volume from muscle origin to insertion is the phenomenon of tumour foci extending far beyond the pseudocapsule of the tumour and the observation that fascial planes act as natural barriers to spread.[20] In reality the incidence of distant satellite lesions is probably small.

Our study originated as an audit, undertaken to see if changes could be made to reduce local recurrence. However, retrospective assessment of the gross tumour volume (GTV), planning target volume (PTV) and relative site of recurrence proved difficult. In particular it was difficult to assess the relationship between GTV and PTV in three dimensions, even when all the written and radiographic records were available.

Whether treatment of the entire muscle compartment is necessary has been called into question by Mundt's data[7] which showed no benefit in treating patients with an initial field margin of greater than 10 cm. To date there has been no prospective evaluation of the radiotherapy margins in the post-operative treatment of soft tissue sarcoma. Similarly, dose has not been assessed in a prospective study. Whether there is a sufficient number of patients for a successful randomised trial is questionable, particularly in view of the inconsistency in surgical management and the variety of tumour-related factors.

An alternative would be to establish a means of prospectively reporting all cases treated with combined modality treatment in which would be recorded:

1. Status of the surgical margins.
2. GTV, clinical target volume (CTV) and PTV described in a standardised manner with minimum margins in three dimensions.
3. Dose delivered to the PTV expressed in accordance with ICRU guidelines.
4. Standardised late radiation morbidity score.
5. Relationship of site of recurrence to the phase 1 and 2 PTV confirmed by re-simulation.

In the absence of an evidence base this would provide a starting point towards a standard treatment policy across centres, allowing for the prospective assessment of field margins or even a randomised trial. Given the evidence that late morbidity is related to both the volume irradiated and the total dose,[8,11] there is clearly a need to optimise the dimensions of the irradiated volume to achieve the best therapeutic ratio. Furthermore, there are undoubtedly patients who do not require post-operative radiotherapy.[17] In the future it may be possible to predict with more accuracy those patients who will be controlled locally by surgery alone and who therefore may be spared the morbidity of radiotherapy.

### Conclusions

The greatest challenge in reducing local recurrence of high-grade sarcomas by adjuvant radiotherapy is the control of microscopic disease in the tumour bed. Irradiation of the entire muscle compartment may not be necessary. However, caution should be exercised when using a retrospective review of this kind to recommend changes in treatment policy. We have highlighted the need for accurate recording of the site and dimensions of the primary tumour and the radiotherapy parameters employed. If the precise relationship between site of recurrence and irradiated volume as well as treatment morbidity are prospectively recorded, then it may be possible to define an evidence based optimal treatment policy.

### Acknowledgements

We would like to thank all the surgeons who have been kind enough to refer patients for radiotherapy, in particular Mr Meirion Thomas. We are very grateful to colleagues in the departments of Physics, Radiotherapy and Radiology and to Dr Cyril Fisher in the Department of Histopathology.

### References

1 Leibel SA, Tranbaugh RF, Wara WM, Beckstead JH, Bovill EG, Phillips TL. Soft tissue sarcoma of the extremities. *Cancer* 1982; 50:1076–83.
2 Lindberg RD, Martin RG, Romsdahl MM, Barkley HT. Conservative surgery and postoperative radiotherapy in 300 adults with soft-tissue sarcomas. *Cancer* 1981; 47(5):2391–7.
3 Robinson M, Barr L, Fisher C, Fryatt I, Stotter A, Harmer C, Wiltshaw E, Westbury G. Treatment of extremity soft tissue sarcomas with surgery and radiotherapy. *Radiother Oncol* 1990; 18:221–33.
4 Suit HD, Mankin HJ, Wood WC, Gebhardt MC, Harmon DC, Rosenberg A, Tepper JE, Rosenthal D. Treatment of the patient with stage M0 soft tissue sarcoma. *J Clin Oncol* 1988; 6(5):854–62.
5 Rosenberg, SA, Tepper J, Glatstein E, Costa J, Baker A, Brennan M, DeMoss EV, Seipp C, Sindelar WF, Surgarbaker P, Wesley R. The treatment of soft-tissue sarcomas of the extremities. *Ann Surg* 1982; 196:305–14.
6 Fein DA, Lee WR, Lanciano RM, Corn BW, Herbert SH, Hanlon AL, Hoffman JP, Eisenberg BL, Coia LR. Management of extremity soft tissue sarcomas with limb-sparing surgery and postoperative irradiation: do total dose, overall treatment time and the surgical-radiotherapy interval impact on local control? *Int J Radiat Oncol Biol Phys* 1995; 32(4):969–76.
7 Mundt AJ, Awan A, Sibley GS, Simon M, Rubin SJ, Samuels B, Wong W, Beckett M, Vijayakumar, S, Weichselbaum RR. Conservative surgery and adjuvant radiation therapy in the management of adult soft tissue sarcoma of the extremities; clinical and radiobiological results. *Int J Radiat Oncol Biol Phys* 1995; 32(4):977–85.
8 LeVay J, O'Sullivan B, Catton C, Bell R, Fornasier V, Cummings B, Hao Y, Warr D, Quirt I. Outcome and prognostic factors in soft tissue sarcoma in the adult. *Int J Radiat Oncol Biol Phys* 1993; 27:1091–9.
9 Simon MA, Enneking WF. The management of soft-tissue sarcomas of the extremity. *J Bone Joint Surg* 1976; 58A(3):317–27.
10 Shiu MH, Castro EB, Hajdu SI, Fortner J. Surgical treatment of 297 soft tissue sarcomas of the lower extremity. *Ann Surg* 1975; 182:597–602
11 Stinson,SF, DeLaney TF, Greenberg J, Yang JC, Lampert MH, Hicks JE, Venzon D, White DE, Rosenderg SA, Glatstein EJ. Acute and long-term effects on limb function of combined modality limb sparing therapy for extremity soft tissue sarcoma. *Int J Radiat. Oncol Biol Phys* 1991; 21:1493–9.
12 Pisters PW, Harrison LB, Leung DH, Woodruff JM, Casper ES, Brennan MF. Long-term results of a prospective randomized trial of adjuvant brachtherapy in soft tissue sarcoma. *J Clin Oncol* 1996; 14(3):859–68.
13 Harmer C. Management of soft tissue sarcomas. In: Selby P, Bailey C, eds. *Cancer and the Adolescent*. London: BMJ Publishing Group, 1996:69–89.
14 Sobin LH, Wittekind Ch. UICC TMN Classification of Malignant Tumours. New York: Wiley–Liss, 1997.
15 Harmer CL, Bidmead M. Three-dimensional planning and conformal radiotherapy. In: Verweij J, Pinedo HM, Suit HD, eds. *Soft Tissue Sarcomas: Present Achievements and Future Prospects*. Dordrecht: Kluwer, 1997:129–41.
16 Jacob R, Gilligan D, Robinson M, Harmer C. Hyperfractionated radiotherapy for soft tissue sarcoma. *Sarcoma* 1999; 3:157–65.
17 Yang J, Chang A, Baker A. Randomised prospective study of the benefit of adjuvant radiation therapy in the treatment of soft tissue sarcomas of the extremity. *J Clin Oncol* 1998; 16(1):197–203.
18 Pisters P, Leung D, Woodruff J, Shi W. Analysis of prognostic factors in 1,041 patients with localized soft tissues sarcoma of the exremities. *J Clin Oncol* 1996; 14(5):1679–89.
19 Hall EJ. *Radiobiology for the Radiologist*. Philadelphia: JB Lippincott, 1994
20 Westbury G. Surgery in the management of soft tissue sarcoma. *Clin Oncol* 1989; 1:101–5.
